# Uterine Hemostatics

**Published:** 1884-05

**Authors:** J. Braxton Hicks


### Selections.

### Uterine Hemostatics.

As a small contribution to the practical portion of the subject of uterine hemostatics, I venture to make a few remarks on the mechanical kinds, which we know by the name of plugs or tents. In doing so I must be understood to refer only to those cases where the cavity of the uterus is not sufficiently large to contain blood in quantity, the loss of which from the circulation is likely to produce anything of serious detriment.

If we go back to former practice and to tex'-books, we find it recommended that in case of threatened abortion with much hemorrhage, a vaginal plug should be used. The vaginal plugs recommended are the tampon, cotton or wool, silk or cambric handkerchief, rags or sponges passed in till the vagina is filled up. An India-rubber ball also has been suggested, covered with felt or such like material. Now, even with the best of management, there is much of distress to the patient in the use of the vaginal plug, and, with regard to its hemostatic effect, very much uncertainty, and generally partial failure, and in the hands of the unskillful and careless, there is positively no restraint of bleeding worth the mention. If at any time any good results be produced, it is rather by the reflex irritation that it causes, whereby the uterus expels its contents. It is not so very rare an occurrence that one finds, on removal of the plug, the ovum on the uppermost part of it. But, besides its palpable inefficiency, a vaginal plug, being of a porous texture, absorbs a large quantity of blood, and thus conceals it from our sight. It also favors decomposition, and this, as is well known, occurs within a few hours, and thus we have a new element of danger.

Again, in many cases, when called to such a case, we have no speculum at hand, and although we may extemporize one out of card-board, book covers, or such like material, yet, before we have thoroughly and firmly filled the vagina we must have given the patient considerable pain and distress, besides having occasion to put such pressure on the urethra as may necessitate subsequent catheterism. For these reasons, namely, the imperfection of action, pain in introduction, and danger if left in long—in other words, its general crudity—it seems to me that as a general rule the vaginal plug should, in the cases I have supposed, be discarded. And as a substitute I would urge the employment of the cervical plug as being more precise in action, as well as being capable, if we use a dilating kind, of expanding the canal for the purpose of exploration, or for the expulsion or removal of its contents.

If, then, in any case of uterine hemorrhage where we have the conditions above alluded to, we desire, besides immediately checking the bleeding, to dilate, we can use the compressed sponge-tent, the best form of which I have found to be those made after Sir James Simpson's plan, by Duncan, Flockhart & Co., Edinburgh. These can be introduced by a long pair of forceps, and retained *in situ* by placing a piece of sponge, with tape attached, in the upper vagina. Of course, even these materials retain some secretions, etc., and tend to facilitate decomposition; but their removal and cleansing can be effected much more readily than the vaginal plug, because it requires but a small portion. The sea-tangle tent, by reason of its slipperiness, is unreliable as a plug in hemorrhage. If we desire, however, only to plug the cervix, we can very easily extemporize a plug from materials to be found in every house. For instance, take a stick (say a flower stick) about a foot long, and taper it at one end to about the size of a uterine sound, or larger; wind round this end, for about three inches down, strips of cambric rag, lint or sponge to the required thickness, judging from the size of the os. Strips of sponge can be readily obtained from the cup-shaped sponges of compact texture, and they can be tied on by thread, layer after layer, till the requisite conical form is obtained. The strips of the other material can be laid on similarly. After the covered end has been well greased it is passed into the canal and the stick retained *in situ*, after the manner in which we tie in a catheter; an elastic tape, if obtainable, is to be preferred.

A catheter or bougie, at the end of a long injection tube, can be treated in the same way. If we require great precision of application, then it is best that the hand should hold the external end till the hemorrhage has ceased. If the catheter and stilet be used, then I have found it convenient to bend the external portion backward, between the buttocks, tying the tape around the ring of the stilet—the ends of the tape being carried, as usual, to back and front of the waist-band.

These more homely adaptations I have recommended, rather than the especially made kinds, because they are often wanted at times when we cannot send home for a showy sort. In any case, a cervical plug, expanding or not, is more precise, less crude and painful in application, than the vaginal, and, in my experience, nearly always successful. In all cases of abortion, where a plug is necessary, I would lay it down as a rule that the expanding tent should be employed. In case of flexion with abortion (and it is this complication which so frequently increases the hemorrhage) it will be found that the covered stick or stemmed plug, above described, is very useful, for, if the fundus be elevated during its introduction, the uterine cavity is straightened and evacuation of the contents thereby facilitated. —*J. Braxton Hicks, M. D., F. R. S., in British Medical Journal.*

